# Rates of serious clinical outcomes in survivors of hospitalisation with COVID-19 in England: a descriptive cohort study within the OpenSAFELY platform

**DOI:** 10.12688/wellcomeopenres.17735.1

**Published:** 2022-04-29

**Authors:** The OpenSAFELY Collaborative, John Tazare, Alex J. Walker, Laurie A. Tomlinson, George Hickman, Christopher T. Rentsch, Elizabeth J. Williamson, Krishnan Bhaskaran, David Evans, Kevin Wing, Rohini Mathur, Angel YS. Wong, Anna Schultze, Seb Bacon, Chris Bates, Caroline E. Morton, Helen J. Curtis, Emily Nightingale, Helen I. McDonald, Amir Mehrkar, Peter Inglesby, Simon Davy, Brian MacKenna, Jonathan Cockburn, William J. Hulme, Charlotte Warren-Gash, Ketaki Bhate, Dorothea Nitsch, Emma Powell, Amy Mulick, Harriet Forbes, Caroline Minassian, Richard Croker, John Parry, Frank Hester, Sam Harper, Rosalind M. Eggo, Stephen JW. Evans, Liam Smeeth, Ian J Douglas, Ben Goldacre

**Affiliations:** 1 London School of Hygiene & Tropical Medicine, London, WC1E 7HT, UK; 2 The DataLab, Nuffield Department of Primary Care Health Sciences, University of Oxford, Oxford, OX26GG, UK; 3 TPP, TPP House, 129 Low Lane, Horsforth, Leeds, LS18 5PX, UK; 4 NIHR Health Protection Research Unit (HPRU) in Immunisation, London, UK

**Keywords:** Electronic Health Records, Covid-19, Pneumonia, Cardiometabolic, Post-hospitalisation

## Abstract

**Background: **Patients surviving hospitalisation for COVID-19 are thought to be at high risk of cardiometabolic and pulmonary complications, but quantification of that risk is limited. We aimed to describe the overall burden of these complications in people after discharge from hospital with COVID-19.

**Methods: **Working on behalf of NHS England, we used linked primary care records, death certificate and hospital data from the OpenSAFELY platform. We constructed three cohorts: patients discharged following hospitalisation with COVID-19, patients discharged following pre-pandemic hospitalisation with pneumonia, and a frequency-matched cohort from the general population in 2019. We studied seven outcomes: deep vein thrombosis (DVT), pulmonary embolism (PE), ischaemic stroke, myocardial infarction (MI), heart failure, AKI and new type 2 diabetes mellitus (T2DM) diagnosis. Absolute rates were measured in each cohort and Fine and Gray models were used to estimate age/sex adjusted subdistribution hazard ratios comparing outcome risk between discharged COVID-19 patients and the two comparator cohorts.

**Results: **Amongst the population of 77,347 patients discharged following hospitalisation with COVID-19, rates for the majority of outcomes peaked in the first month post-discharge, then declined over the following four months. Patients in the COVID-19 population had markedly higher risk of all outcomes compared to matched controls from the 2019 general population. Across the whole study period, the risk of outcomes was more similar when comparing patients discharged with COVID-19 to those discharged with pneumonia in 2019, although COVID-19 patients had higher risk of T2DM (15.2 versus 37.2 [rate per 1,000-person-years for COVID-19 versus pneumonia, respectively]; SHR, 1.46 [95% CI: 1.31 - 1.63]).

**Conclusions: **Risk of cardiometabolic and pulmonary adverse outcomes is markedly raised following discharge from hospitalisation with COVID-19 compared to the general population. However, excess risks were similar to those seen following discharge post-pneumonia. Overall, this suggests a large additional burden on healthcare resources.

## Introduction

Cardiometabolic and pulmonary complications, especially thrombotic events, have been described as a key feature of the severe acute phase of COVID-19. A recent systematic review estimated the risk of venous thromboembolism (VTE) to be ~15% in hospitalised COVID-19 patients, with higher risks observed in people admitted to intensive care (~30% ^1– 3^). Underlying reasons for this higher risk are likely to be multifactorial, including immobility following illness/hospitalisation as well as the known association with infection in general, mediated through interactions with general inflammatory and other immune pathways ^4^. The possibility that SARS-CoV-2 may directly trigger pulmonary thrombi via vascular damage and inflammatory effects in the lung has also been raised ^5^.

As the COVID-19 pandemic has progressed, it is increasingly reported that some patients who recover from the acute disease phase go on to experience a range of post-recovery clinical problems. This post-acute COVID-19 syndrome is currently not well described or understood, with the UK National Institute for Health and Care Excellence stating that any body system could be affected, for an undetermined period of time ^6^. Any such syndrome now needs to be defined and quantified so that patients and health services can know what outcomes may be expected, and plan accordingly ^6, 7^. It is also unclear whether COVID-19 is exceptional in its association with cardiometabolic events, or comparable to other respiratory pathogens, such as influenza and Streptococcus pneumoniae, which have well-described associations with acute cardiovascular events.

Work to date on cardiometabolic outcomes with COVID-19 has largely focused on risks during hospitalisation, for example, highlighting increased myocardial involvement and acute kidney injury complications ^8– 10^. However, there is a lack of evidence on how these risks evolve in survivors of severe COVID-19. We therefore measured the rates of cardiometabolic outcomes in people in England who were discharged from hospital following the acute phase of COVID-19. For context, we compared these rates with those seen prior to the pandemic in both the general population and amongst people discharged following hospitalization for non-COVID-19 pneumonia.

## Methods

### Study design and data sources

We conducted an observational cohort study using electronic health record (EHR) data from primary care practices using TPP software. TPP provides the software to approximately 40% of English general practices and it is used to record all clinical information such as diagnoses, blood tests and prescriptions. This is additionally linked to Office for National Statistics (ONS) death registrations and Secondary Uses Service (SUS) data (containing hospital records) through OpenSAFELY. This is an open-source data analysis platform developed during the COVID-19 pandemic, on behalf of NHS England, to allow near real-time analysis of pseudonymised primary care records at scale, currently covering approximately 40% of the population in England, operating within the EHR vendor’s highly secure data centre ^11, 12^. Details on Information Governance for the OpenSAFELY platform can be found in the Software and reproducibility section.

### Population

We included all adults aged ≥18 years registered with a general practice for ≥1 year on the index date with information on age, sex, and socioeconomic status. From this source population we selected three cohorts: all patients hospitalised with COVID-19 (in the year starting 1 ^st^ February 2020), a comparison cohort containing all patients hospitalised with non-COVID pneumonia across an equivalent period starting in 2019 (i.e. the year starting 1 ^st^ February 2019) and a general population frequency matched cohort in 2019. The COVID-19 and pneumonia cohorts were selected as anyone hospitalised with an associated diagnostic code for COVID-19 or pneumonia respectively (referred to as the “index hospitalisation”). The general population cohort was formed by matching each patient in the COVID-19 cohort to up to five patients eligible on 1st February 2019 in TPP on age (within 1 year), sex and region defined by Sustainability Transformation Partnership level (a more granular form of England NHS region). Matching was performed using a greedy matching algorithm, with no replacement (for more details, see GitHub).

### Outcomes and follow-up

We measured seven outcomes: deep vein thrombosis (DVT), pulmonary embolism (PE), ischaemic stroke, myocardial infarction (MI), heart failure, AKI and new type 2 diabetes mellitus (T2DM) diagnosis.

The study periods ran for one year, starting 1st February in either 2019 or 2020, depending on the population (as defined above). The follow-up period began on the discharge date of the index COVID-19 or pneumonia hospital stay or 1st February 2019 in the general population matched cohort. For each analysis, follow-up ended on the earliest of: the first recorded outcome event, the study end date, or the date of death of the patient. For the AKI outcome, we excluded patients who were receiving dialysis before the index date (defined as presence of a dialysis code or eGFR < 15ml/min). For diabetes, we excluded any patients who had a previous diabetes event, to ensure only incident diagnoses were measured.

Outcomes were defined primarily as the presence of a diagnostic code for each of the respective outcomes, either in the general practice record, in hospital, or as a cause of death on a death certificate. For the primary analysis, we excluded records where the patient had a recent outcome recorded within three months before the index date (including if they had been recorded during the index hospitalisation). This was to prevent double counting of the same event, for example where a general practice (GP) updates the record of a patient, recording an event that occurred during a recent hospitalisation. Sensitivity analyses explored the effect of including these events (see Statistical Methods).

### Statistical methods

We described the characteristics of the three patient cohorts formed: patients discharged from an admission with COVID-19, patients discharged from an admission with pneumonia and patients from the general population matched on age, sex and region. Additionally, for the hospital cohorts, we described the characteristics of the admitted populations to highlight potential differences in those surviving to discharge.

Rates were reported for each outcome, per 1,000 person years, initially for the whole follow-up time, then stratified into time windows: 0–29 days, 30–59 days, 60–89 days, 90120 days and 120+ days post discharge for the COVID-19 and pneumonia cohorts, to determine how the rate of outcomes changed over time. Rates in all three patient cohorts were stratified by age, sex and ethnicity.

We used Fine and Gray regression models to estimate subdistribution hazard ratios (SHRs) and 95% confidence intervals (CIs) to compare the risk of each outcome between, 1) the discharged COVID-19 group and the matched general population group, and 2) the discharged COVID-19 group and the discharged pneumonia group. In the primary analysis, we investigated crude univariable and age and sex adjusted models. The same patient can contribute person-time to all exposure groups, however these periods are non-overlapping therefore we applied robust standard errors. Any evidence of deviations from proportional subdistribution hazards were further investigated using time-period specific SHRs ^13, 14^.

In sensitivity analyses we tested the effect of including 1) previously omitted outcomes recorded in the primary care record when there was a recorded outcome within 3 months before the index date, and 2) only events recorded in hospital or as a cause of death on a death certificate.

Finally, in post-hoc analyses we investigated the robustness of results to those obtained from a model further adjusting for comorbidities and available lifestyle information.

### Software and reproducibility

Data management was performed using the OpenSAFELY software, Python 3.8 and SQL, and analysis using Stata 16.1. All codelists alongside code for data management and analyses can be found in our GitHub repository. All software for the OpenSAFELY platform is available for review and re-use at our organisation GitHub.

NHS England is the data controller; TPP is the data processor; and the key researchers on OpenSAFELY are acting on behalf of NHS England. OpenSAFELY is hosted within the TPP environment which is accredited to the ISO 27001 information security standard and is NHS IG Toolkit compliant ^15, 16^. Patient data are pseudonymised for analysis and linkage using industry standard cryptographic hashing techniques. All pseudonymised datasets transmitted for linkage onto OpenSAFELY are encrypted and access to the platform is via a virtual private network connection, restricted to a small group of researchers who hold contracts with NHS England and only access the platform to initiate database queries and statistical models. All database activity is logged; only aggregate statistical outputs leave the platform environment following best practice for anonymisation of results such as statistical disclosure control for low cell counts ^17^. The OpenSAFELY research platform adheres to the obligations of the UK General Data Protection Regulation (GDPR) and the Data Protection Act 2018. In March 2020, the Secretary of State for Health and Social Care used powers under the UK Health Service (Control of Patient Information) Regulations 2002 (COPI) to require organisations to process confidential patient information for the purposes of protecting public health, providing healthcare services to the public and monitoring and managing the COVID-19 outbreak and incidents of exposure; this sets aside the requirement for patient consent ^18^. Taken together, these provide the legal bases to link patient datasets on the OpenSAFELY platform.

This study was approved by the Health Research Authority (REC reference 20/LO/0651) and by the LSHTM Ethics Board (ref 21863).

## Results

We identified 77,347 patients discharged following an admission with COVID-19 in the year starting from 1 ^st^ February 2020 and 127,987 patients discharged with pneumonia in the year starting from 1 ^st^ February 2019. For each patient discharged following an admission with COVID-19 we matched up to five patients from the general population eligible in TPP on 1st February 2019 on age, sex and region. We successfully matched five patients for over 99.9% of COVID-19 patients.

We present characteristics for the cohorts studied in Table 1 and Table 2. Compared to the discharged COVID-19 cohort, the discharged pneumonia cohort had a higher proportion aged over 80. The ethnic breakdown was broadly similar between the three groups, although the discharged COVID-19 patients had a higher proportion of patients who were Asian and Asian British. Furthermore, Table 1 highlights that the discharged pneumonia patients had a higher proportion of comorbidities (including history of the investigated outcomes) compared to the discharged COVID-19 and general population cohorts.

**Table 1.** Patient characteristics amongst patients discharged with COVID-19 (in the year starting from 1 ^st^ February 2020), all patients discharged with non-COVID pneumonia (in the year starting from 1 ^st^ February 2019) and a matched (age, sex and region) general population comparator group.

Characteristic (%)	Discharged Pneumonia (2019)	Matched General Population (2019)	Discharged COVID-19
Total	127987	386669	77347
Age			
Median	76	68	68
18 – 49	12847 (10.0)	78200 (20.2)	15640 (20.2)
50 – 59	11652 (9.1)	59878 (15.5)	11976 (15.5)
60 – 69	19320 (15.1)	62862 (16.3)	12573 (16.3)
70 – 79	32686 (25.5)	77979 (20.2)	15598 (20.2)
80 +	51482 (40.2)	107750 (27.9)	21560 (27.9)
Sex			
Male	64347 (50.3)	199013 (51.5)	39808 (51.5)
Female	63640 (49.7)	187656 (48.5)	37539 (48.5)
Ethnicity			
White	85927 (67.1)	249671 (64.6)	48700 (63.0)
Mixed	471 (0.4)	2511 (0.6)	716 (0.9)
Asian /Asian British	4522 (3.5)	19528 (5.1)	753 (9.8)
Black	1210 (0.9)	6717 (1.7)	2195 (2.8)
Other	728 (0.6)	4667 (1.2)	1187 (1.5)
Unknown	35129 (27.4)	103575 (26.8)	16976 (21.9)
Body mass index (kg/m ^2^)			
Not obese	92424 (72.2)	293263 (75.8)	48415 (62.6)
30-34.9 (Obese class I)	21415 (16.7)	63349 (16.4)	16386 (21.2)
35-39.9 (Obese class II)	9064 (7.1)	21051 (5.4)	7750 (10.0)
≥40 (Obese class III)	5084 (4.0)	9006 (2.3)	4796 (6.2)
Smoking Status			
Never	37684 (29.4)	172500 (44.6)	31850 (41.2)
Former	67638 (52.8)	166214 (43.0)	38441 (49.7)
Current	22665 (17.7)	47955 (12.4)	7056 (9.1)
Index of Multiple Deprivation			
1 (least deprived)	25788 (20.1)	77594 (20.1)	15576 (20.1)
2	25656 (20.0)	78028 (20.2)	15377 (19.9)
3	25555 (20.0)	77010 (19.9)	15561 (20.1)
4	25684 (20.1)	77054 (19.9)	15442 (20.0)
5 (most deprived)	25304 (19.8)	76983 (19.9)	15391 (19.9)
**Comorbidities**			
Hypertension	72497 (56.6)	160834 (41.6)	38508 (49.8)
Chronic respiratory disease	37418 (29.2)	31526 (8.2)	12431 (16.1)
Asthma			
With no oral steroid use	102662 (80.2)	333549 (86.3)	62628 (81.0)
With oral steroid use	18063 (14.1)	45910 (11.9)	11360 (14.7)
Chronic heart disease	43369 (33.9)	61886 (16.0)	19295 (24.9)
Diabetes			
With HbA1c≥58 mmol/mol	23960 (18.7)	46677 (12.1)	14221 (18.4)
With HbA1c<58 mmol/mol	11735 (9.2)	16809 (4.3)	8913 (11.5)
With no recent HbA1c measure	2100 (1.6)	3888 (1.0)	1498 (1.9)
Cancer (non-haematological)			
Diagnosed <1 year ago	6637 (5.2)	3512 (0.9)	2079 (2.7)
Diagnosed 1-4.9 years ago	6464 (5.1)	9463 (2.4)	2585 (3.3)
Diagnosed ≥ 5 years ago	12847 (10.0)	25033 (6.5)	5785 (7.5)
Haematological malignancy			
Diagnosed <1 year ago	1067 (0.8)	379 (0.1)	370 (0.5)
Diagnosed 1-4.9 years ago	1419 (1.1)	1201 (0.3)	513 (0.7)
Diagnosed ≥ 5 years ago	2223 (1.7)	2459 (0.6)	813 (1.1)
Reduced kidney function			
Estimated GFR 30–60	34228 (26.7)	66214 (17.1)	15876 (20.5)
Estimated GFR 15–29	5312 (4.2)	5338 (1.4)	2262 (2.9)
Estimated GFR < 15 or dialysis	1925 (1.5)	778 (0.2)	1086 (1.4)
Chronic Liver Disease	3169 (2.5)	2677 (0.7)	1559 (2.0)
Dementia	13520 (10.6)	16968 (4.4)	6753 (8.7)
Other neurological disease	5714 (4.5)	5987 (1.5)	2965 (3.8)
Asplenia	759 (0.6)	803 (0.2)	347 (0.4)
Rheum arthritis/lupus/psoriasis	12619 (9.9)	23762 (6.1)	6413 (8.3)
Other immunosuppressive disease	643 (0.5)	900 (0.2)	325 (0.4)
**History of**			
DVT	8452 (6.6)	9459 (2.4)	4080 (5.3)
Stroke	15547 (12.1)	16394 (4.2)	7640 (9.9)
PE	9439 (7.4)	6229 (1.6)	5264 (6.8)
MI	15922 (12.4)	18835 (4.9)	6882 (8.9)
AKI	39614 (31.0)	14449 (3.7)	19722 (25.5)
Heart failure	35233 (27.5)	22784 (5.9)	13193 (17.1)

**Table 2.** Patient characteristics amongst patients admitted with COVID-19 and non-COVID pneumonia.

Characteristic (%)	Admitted Pneumonia (2019)	Admitted COVID-19
Total	149597	98209
Age		
Median	77	72
18 – 49	13331 (8.9)	16065 (16.4)
50 – 59	12624 (8.4)	13065 (13.3)
60 – 69	21517 (14.4)	15003 (15.3)
70 – 79	37773 (25.2)	21177 (21.6)
80 +	64352 (43.0)	32899 (33.5)
Sex		
Male	74342 (50.3)	52188 (53.1)
Female	75255 (49.7)	46021 (46.9)
Ethnicity		
White	100284 (67.0)	62449 (63.6)
Mixed	527 (0.4)	820 (0.8)
Asian /Asian British	5104 (3.4)	8917 (9.1)
Black	1343 (0.9)	2588 (2.6)
Other	825 (0.6)	1353 (1.4)
Unknown	41514 (27.8)	22082 (22.5)
Body mass index (kg/m ^2^)		
Not obese	109483 (73.2)	62613 (63.8)
30–34.9 (Obese class I)	24323 (16.3)	20399 (20.8)
35–39.9 (Obese class II)	10125 (6.8)	9444 (9.6)
≥40 (Obese class III)	5666 (3.8)	5753 (5.9)
Smoking Status		
Never	43727 (29.2)	38209 (38.9)
Former	80018 (53.5)	51305 (52.2)
Current	25852 (17.3)	8695 (8.9)
Index of Multiple Deprivation		
1 (least deprived)	25788 (20.3)	19709 (20.1)
2	29633 (19.8)	19695 (20.1)
3	30339 (20.3)	19739 (20.1)
4	29584 (19.8)	19669 (20.0)
5 (most deprived)	29744 (19.9)	19397 (19.8)
**Comorbidities**		
Hypertension	86795 (58.0)	52456 (53.4)
Chronic respiratory disease	43816 (29.3)	17521 (17.8)
Asthma		
With no oral steroid use	121227 (81.0)	80159 (81.6)
With oral steroid use	20357 (13.6)	13901 (14.2)
Chronic heart disease	52405 (35.0)	27669 (28.2)
Diabetes		
With HbA1c·58 mmol/mol	28511 (19.1)	19332 (19.7)
With HbA1c<58 mmol/mol	13725 (9.2)	11783 (12.0)
With no recent HbA1c measure	2474 (1.7)	1920 (2.0)
Cancer (non-haematological)		
Diagnosed <1 year ago	8159 (5.5)	2774 (2.8)
Diagnosed 1-4.9 years ago	7923 (5.3)	3608 (3.7)
Diagnosed ≥ 5 years ago	15477 (10.3)	8163 (8.3)
Haematological malignancy		
Diagnosed <1 year ago	1320 (0.9)	514 (0.5)
Diagnosed 1-4.9 years ago	1714 (1.1)	766 (0.8)
Diagnosed ≥ 5 years ago	2628 (1.8)	1208 (1.2)
Reduced kidney function		
Estimated GFR 30–60	41764 (27.9)	23437 (23.9)
Estimated GFR 15–29	7091 (4.2)	3845 (3.9)
Estimated GFR < 15 or dialysis	2402 (1.6)	1581 (1.6)
Chronic Liver Disease	3840 (2.6)	2101 (2.1)
Dementia	16627 (11.1)	10297 (10.5)
Other neurological disease	6771 (4.5)	4182 (4.3)
Asplenia	862 (0.6)	434 (0.4)
Rheum arthritis/lupus/psoriasis	14710 (9.8)	8426 (8.6)
Other immunosuppressive disease	759 (0.5)	409 (0.4)
**History of**		
DVT	9977 (6.7)	5636 (5.7)
Stroke	19344 (12.9)	11094 (11.3)
PE	11199 (7.5)	7053 (7.2)
MI	19805 (13.2)	10192 (10.4)
AKI	51343 (34.3)	30837 (31.4)
Heart failure	44730 (29.9)	20415 (20.8)

By design, this work focuses on describing the burden of disease amongst patients discharged after severe COVID and our aim is not to make causal conclusions surrounding differences in the risk of outcomes. However, for additional context, the characteristics for the admitted COVID-19 and pneumonia populations are provided in Table 2. Comparison between Table 1 and Table 2 highlights the increased in-patient mortality of admitted COVID-19 patients (21%) compared to admitted pneumonia patients (14%). Furthermore, whilst Table 2 highlights expected differences in the age distribution between the admitted and discharged cohorts, the overall pattern of characteristics between the admitted and discharged groups remained largely consistent.

**Figure 1.**
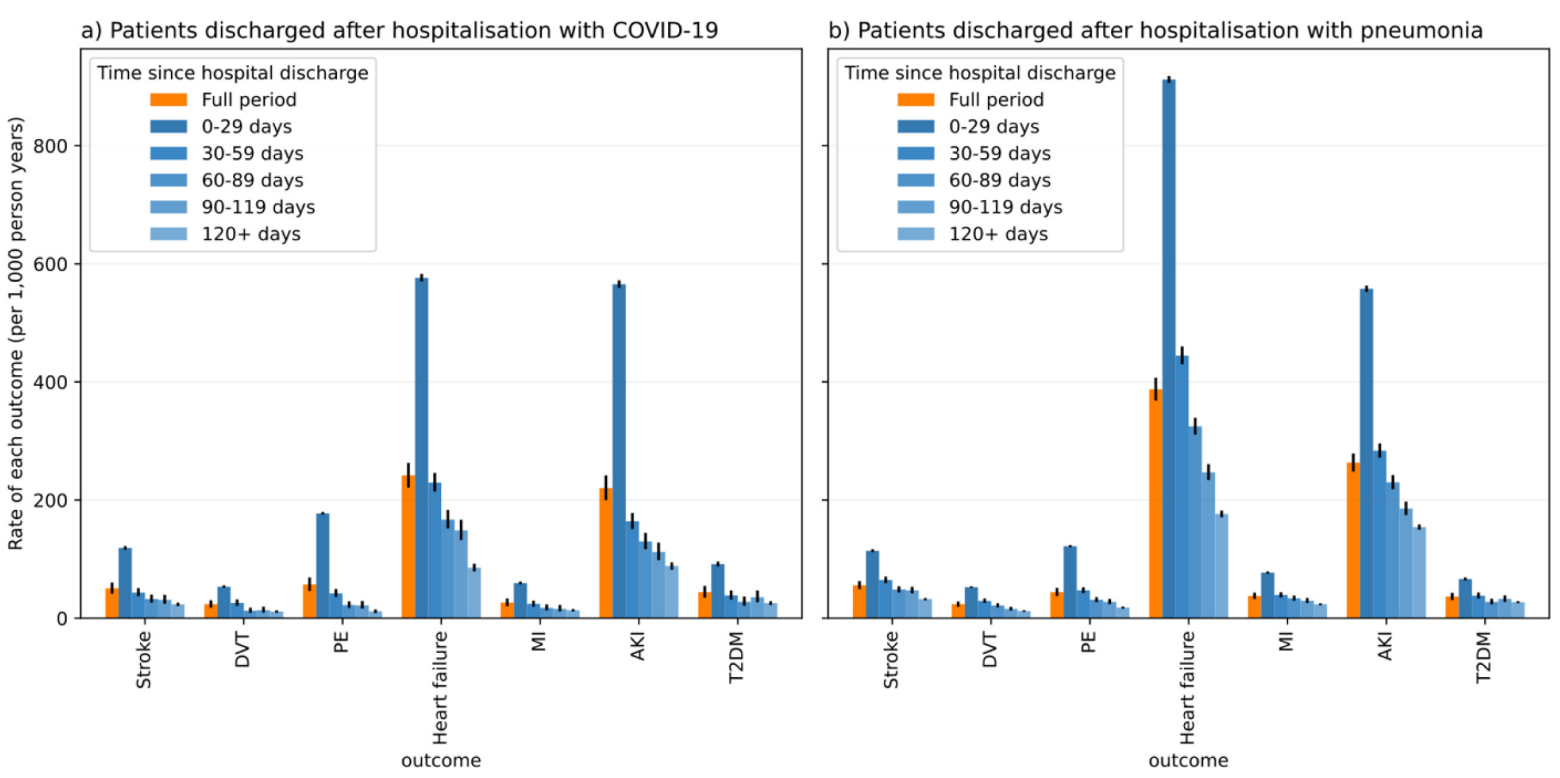
Rate of outcomes (events per 1,000 person years) in time periods following hospital discharge for COVID-19 and pneumonia patients.

Overall rates of each outcome per 1,000 person years for the whole follow-up are presented in Table 3. For the majority of outcomes, we observed higher rates of serious cardiometabolic and pulmonary complications in discharged pneumonia patients compared to both discharged COVID-19 patients and the matched general population group ( Table 3, Figure 1). Across all three cohorts, the largest absolute rates were for AKI and heart failure. Overall rates stratified by age, sex and ethnicity are presented in the Extended Data (Supplemental Tables 5–11).

**Table 3.** Overall rates of outcomes (events per 1,000 person years) amongst patients discharged with COVID-19, all patients discharged with non-COVID pneumonia and a matched (age, sex and region) general population comparator group.

Outcome	Analysis Group	Person time (x 1,000 person years)	Number of events	Rate (95% CI)
Stroke	Discharged COVID-19	22.3	1128	50.5(47.7–53.6)
	General Population	375.9	3938	10.5(10.2–10.8)
	Discharged Pneumonia	52.6	2939	55.8(53.9–57.9)
DVT	Discharged COVID-19	22.3	529	23.7(21.8–25.8)
	General Population	376.5	1357	3.6(3.4–3.8)
	Discharged Pneumonia	52.7	1248	23.7(22.4–25.0)
PE	Discharged COVID-19	22.2	1270	57.2(54.1–60.4)
	General Population	376.6	1327	3.5(3.3–3.7)
	Discharged Pneumonia	52.5	2316	44.1(42.3–45.9)
Heart Failure	Discharged COVID-19	21.3	5152	241.7(235.2–248.4)
	General Population	371.1	14123	38.1(37.4–38.7)
	Discharged Pneumonia	47.9	18566	387.5(381.9–393.1)
MI	Discharged COVID-19	22.4	593	26.5(24.5–28.7)
	General Population	376.1	2797	7.4(7.2–7.7)
	Discharged Pneumonia	52.7	1985	37.7(36.0–39.3)
AKI	Discharged COVID-19	21.2	4522	213.0(206.9–219.3)
	General Population	373	10277	27.6(27.0–28.1)
	Discharged Pneumonia	49.2	12631	256.6(252.1–261.1)
T2DM	Discharged COVID-19	15.2	670	44.1(40.9–47.6)
	General Population	310.9	2763	8.9(8.6–9.2)
	Discharged Pneumonia	37.1	1360	36.6(34.7–38.6)

For COVID-19 patients, rates of stroke were higher amongst the over 80 group compared to the other comparison groups, although the pattern was not consistent across other ages groups. Similarly, rates were not constant by ethnic group, for example, the rate of new T2DM diagnoses were slightly higher amongst black patients discharged following an admission with COVID-19.

Stratified overall rates in 30-day time windows are shown in Figure 1. For discharged COVID-19 patients, we observed the highest rates for all outcomes in the first 30 days post-discharge, with a gradual decline in subsequent periods, consistent with the pattern of rates observed for patients discharged with pneumonia in 2019. For the discharged COVID-19 and pneumonia groups, we observed pronounced rates of AKI and heart failure in the first 30 days post-discharge ( Figure 1).

**Figure 2.**
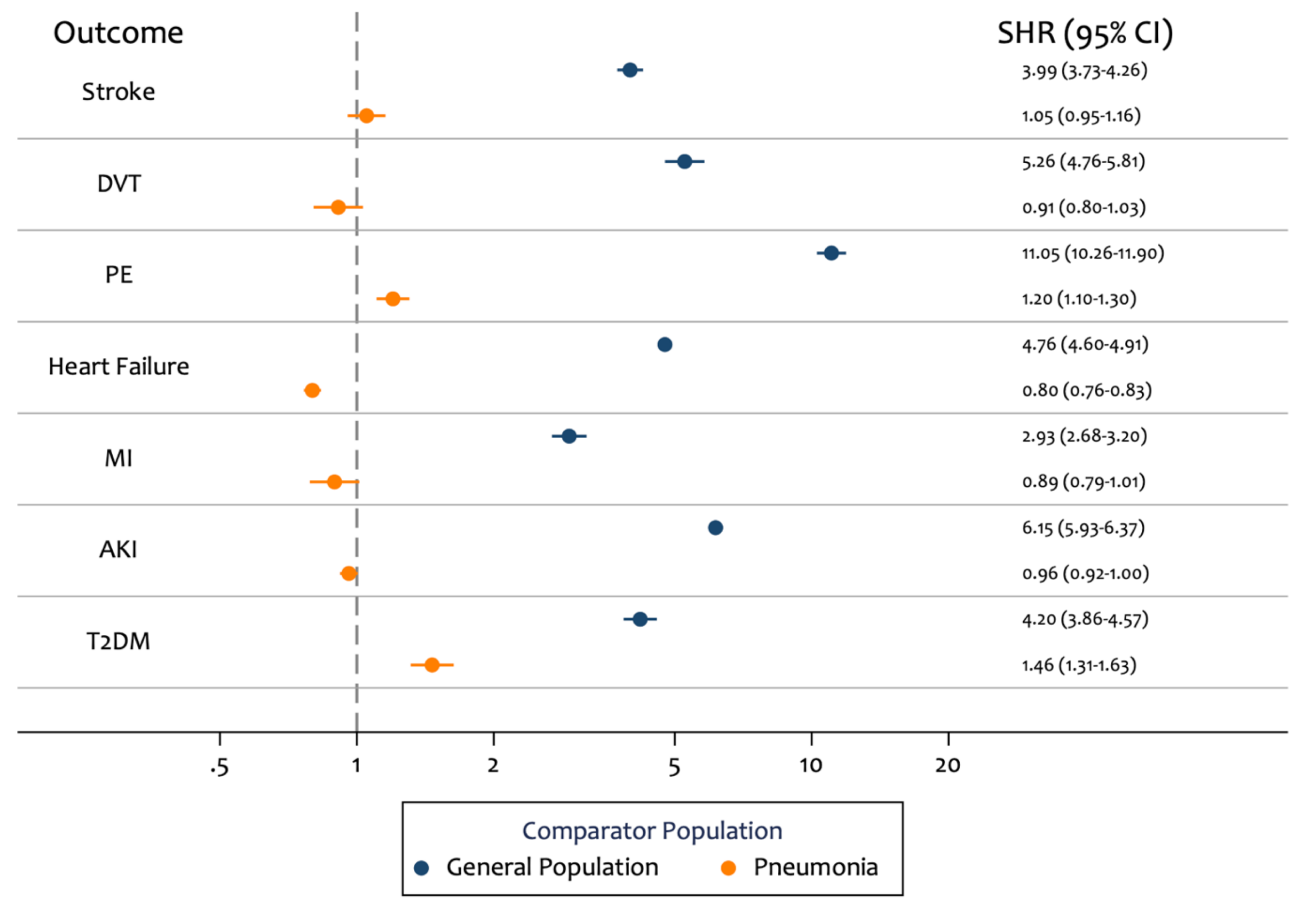
Age and sex adjusted subdistribution hazard ratios (SHRs) for risk of outcomes in patients who were hospitalised with COVID-19 and then discharged, compared to 1) patients who were hospitalised with pneumonia and then discharged, and 2) an age, sex and region matched general population comparator group.

After age and sex adjustment, the discharged COVID-19 group had markedly higher risk for all outcomes compared to the matched general population group ( Table 4, Figure 2). The largest increase in risk was observed for PE (SHR 11.05; 95% CI: 10.26 - 11.90) and AKI (SHR 6.15; 95% CI: 5.93 - 6.37). For the comparison with discharged pneumonia patients, discharged COVID-19 patients had similar risks for the majority of outcomes. We observed higher risk of new T2DM (SHR 1.46; 95% CI: 1.31 - 1.63) and PE (SHR 1.20; 95% CI: 1.10 - 1.30) in patients discharged with COVID-19 compared to patients discharged with pneumonia.

**Table 4.** Estimated subdistribution hazard ratios (SHRs) from univariable, age and sex adjusted, and post-hoc fully-adjusted Fine and Gray models comparing the risk of outcomes in patients who were hospitalised with COVID-19 and then discharged, compared to 1) patients who were hospitalised with pneumonia and then discharged, and 2) an age, sex and region matched general population comparator group. Person time is reported per 1,000 person years.

Outcome	Comparator	Comparator Person Time	Comparator Number of events	Discharged COVID-19 Person Time	Discharged COVID-19 Number of events	Crude SHR (95% CI)	Age/Sex adjusted SHR (95% CI)	Fully-adjusted SHR (95% CI) *
Stroke	General Population	375.9	3938	22.3	1128	3.97(3.72-4.24)	3.99(3.73-4.26)	2.82(2.62-3.05)
	Pneumonia	52.6	2939	22.3	1128	0.84(0.78-0.90)	1.05(0.95-1.16)	1.03(0.93-1.14)
DVT	General Population	376.5	1357	22.3	529	5.25(4.75-5.80)	5.26(4.76-5.81)	3.84(3.39-4.35)
	Pneumonia	52.7	1248	22.3	529	0.92(0.83-1.02)	0.91(0.80-1.03)	1.03(0.91-1.18)
PE	General Population	376.6	1327	22.2	1270	11.06(10.27-11.92)	11.05(10.26-11.90)	7.64(6.96-8.40)
	Pneumonia	52.5	2316	22.2	1270	1.13(1.05-1.21)	1.20(1.10-1.30)	1.22(1.12-1.33)
Heart Failure	General Population	371.1	14123	21.3	5152	4.61(4.46-4.76)	4.76(4.60-4.91)	2.28(2.19-2.38)
	Pneumonia	47.9	18566	21.3	5152	0.57(0.55-0.59)	0.80(0.76-0.83)	0.86(0.82-0.90)
MI	General Population	376.1	2797	22.4	593	2.94(2.70-3.21)	2.93(2.68-3.20)	1.91(1.72-2.12)
	Pneumonia	52.7	1985	22.4	593	0.66(0.60-0.72)	0.89(0.79-1.01)	0.90(0.79-1.03)
AKI	General Population	373	10277	21.2	4522	6.06(5.85-6.27)	6.15(5.93-6.37)	3.17(3.04-3.32)
	Pneumonia	49.2	12631	21.2	4522	0.77(0.74-0.80)	0.96(0.92-1.00)	0.99(0.95-1.04)
T2DM	General Population	310.9	2763	15.2	670	4.16(3.82-4.52)	4.20(3.86-4.57)	2.71(2.45-2.99)
	Pneumonia	37.1	1360	15.2	670	1.15(1.05-1.26)	1.46(1.31-1.63)	1.30(1.15-1.47)

*Covariates included in the fully adjusted model were: age (parametrised as a four-knot restricted cubic spline), sex, ethnicity, obesity, smoking status, index of multiple deprivation quintile, region, hypertension, asthma, chronic respiratory diseases other than asthma, chronic heart disease, diabetes, non-haematological and haematological cancer, reduced kidney function, chronic liver disease, dementia, other neurological disease, organ transplant, asplenia (splenectomy or a spleen dysfunction, including sickle cell disease), rheumatoid arthritis, lupus, or psoriasis, other immunosuppressive conditions (permanent immunodeficiency ever diagnosed or aplastic anaemia or temporary immunodeficiency recorded within the past year) and any history of the outcomes studied.

Results from a sensitivity analysis investigating robustness of absolute and relative rates to outcome definition are presented in the Extended Data. Change in outcome definitions did not meaningfully alter conclusions.

**Figure 3.**
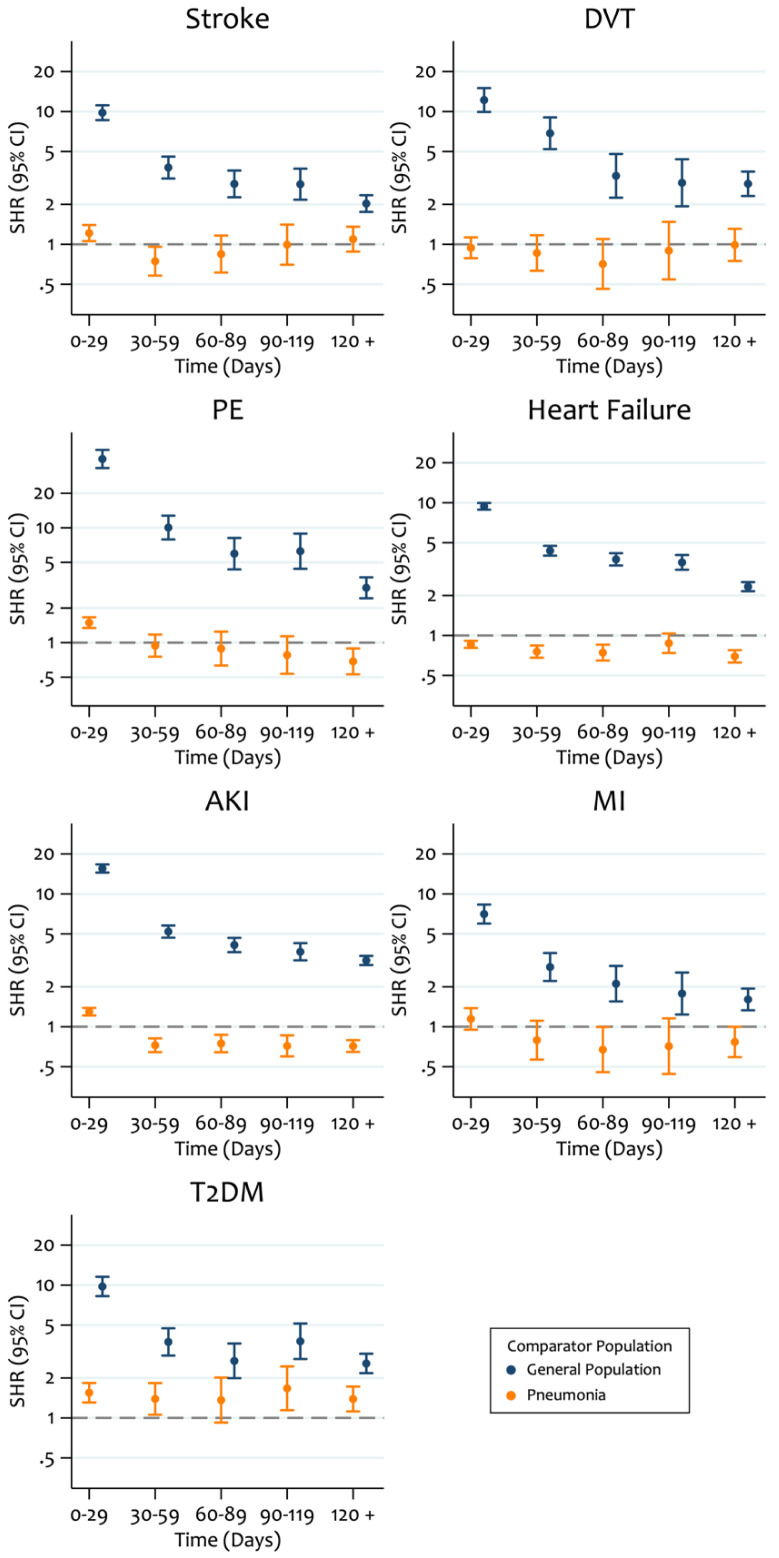
Post-hoc estimated time-period specific subdistribution hazard ratios (SHRs) from age and sex adjusted Fine and Gray models comparing the risk of outcomes in patients who were hospitalised with COVID-19 and then discharged, compared to 1) patients who were hospitalised with pneumonia and then discharged, and 2) an age, sex and region matched general population comparator group.

Finally, given evidence of non-proportional subdistribution hazards we present SHRs in 30-day periods ( Figure 3). For all outcomes, we observed substantially higher SHRs in the first 30-days post-discharge for COVID-19 patients compared to the general population group which gradually reduced in subsequent periods. Furthermore, during the first 30-days post-discharge we observed higher risk of AKI and PE in the discharged COVID-19 group compared to the pneumonia group.

## Discussion

### Key findings

In this descriptive study, we set out to report the overall rates of seven cardiometabolic and pulmonary outcomes in three cohorts: 1) patients discharged following hospitalisation with COVID-19 (in the year from 1 ^st^ Febraury 2020), 2) patients discharged following hospitalisation with pneumonia (in the year from 1 ^st^ February 2019) and 3) a frequency-matched group of patients from the general population in 2019. We found that the rate of cardiometabolic and pulmonary complications following discharge from hospitalisation with COVID-19 was notably higher compared to an age, sex and region matched general population cohort, especially for PE and AKI. However, patients discharged with COVID-19 followed a broadly similar pattern of elevated risk to those discharged from hospital after pneumonia in 2019. In post-hoc analyses, further adjustment for comorbidities and lifestyle information resulted in similar, though slightly attenuated associations ( Table 4).

The pattern of change in the rate of outcomes over time following discharge from hospital was broadly similar between the COVID-19 and pneumonia patients, with the highest rate in the initial 30 days of follow-up, then a 2–3-fold drop in the next 30 days, followed by a more gradual decline. In both discharge-based cohorts, rates remained substantial even after more than 120 days. This highlights an additional short-term and potential long-term burden on healthcare services ^19^.

We did not attempt to determine the casual nature of any observed differences. In the context of the rapidly changing pandemic, we aimed to provide a description of the burden of outcomes after discharge from hospitalisation with COVID-19, compared to cohorts discharged from hospitalisation with pneumonia and the general population, to inform health services and provision. Any study aiming to draw causal conclusions would require careful investigation and adjustment for features of the pathophysiology associated with SARS-CoV-2 infection, changes in healthcare provision during the pandemic and potentially differential likelihood of ascertainment of pre-existing conditions; especially in light of the observed higher in-hospital mortality rate for hospitalised COVID-19 patients compared to patients hospitalised with pneumonia.

### Strengths and limitations

We were able to source our cohorts from the OpenSAFELY platform, which contains data on over 17m adults. This gave us a population who were discharged following hospitalisation with COVID-19 of 77,347, allowing us to obtain precise estimates of the rate of each outcome. We were also able to draw on multiple linked data sources, including primary care records, hospitalisations and death certificates. This allows a more complete picture to be presented of the clinical activity surrounding each outcome.

Much of the research characterising cardiometabolic outcomes in COVID-19 patients to date has focused on in-hospital clinical activity and describing the proportion of events in a narrow time-window post-discharge ^9, 10, 20– 23^. However, our use of both a general population and active control population of patients hospitalised with pneumonia in 2019 provides useful context for the rates of these outcomes in COVID-19 patients who survive hospitalisation. Furthermore, presenting the rates in this context is more informative than within a general population alone and offers an important comparison with a cohort experiencing exposure to another acute respiratory illness event requiring hospitalisation.

Our study aimed to describe clinical events that occurred *after* discharge from hospital, and not the total additional morbidity burden of COVID-19 hospitalisation: specifically, we did not set out to describe events that occurred *during* hospital admission with COVID-19 or pneumonia. However, in our view reliable analysis of in-hospital events may only be achievable with bespoke collections of detailed hospital data, due to shortcomings in routinely collected administrative data that are widely used for such analyses. For example, SUS and HES data contain a list of diagnostic codes associated with each hospitalisation, but they do not contain sufficient information to determine the exact timing of all events within each hospitalisation episode. This means that time-to event analyses are not possible. Similarly, it is not possible to reliably determine the sequence of events during hospitalisation: so, a patient hospitalised with COVID-19, who later had a stroke, may be coded in a similar way to a patient who was hospitalised with a stroke, and then infected with SARS-CoV-2 while in hospital. In addition, routine PCR testing on hospitalised patients during the pandemic may lead to very high ascertainment of infection with SARS-CoV-2, which may not have occurred to the same extent in the comparison population for pneumonia admissions.

It has been reported that there was a marked reduction in GP and hospital activity during the first wave of the pandemic, for example a 40% reduction in admissions for acute coronary syndrome ^24– 26^. This may be in part explained by a reluctance of patients to present at healthcare services for fear of contracting the virus. As a result, we believe population-level rates of many outcomes will be under-ascertained during 2020 compared with 2019. It is unknown whether this applies in the same way to patients who have already had severe COVID-19; if ascertainment is lower, then this would result in a possible under-estimate of outcome rates associated with COVID-19 in our study.

A recent observational study measured similar outcome events in a population of patients discharged from hospital following COVID-19 ^27^. They observed elevated rates in the COVID-19 population compared to a matched general population control group. Our findings are consistent in showing similarly higher rates of outcomes in patients post-discharge with COVID-19. However, importantly we further show that these higher rates of outcomes are broadly comparable, if not slightly lower, when compared to people discharged from hospital following pneumonia, selected as a major non-COVID respiratory infection.

## Conclusion

In this study, the rate of cardiometabolic and pulmonary events in COVID-19 survivors discharged from hospitalisation was elevated in a similar manner to patients discharged from hospitalisation with pre-pandemic pneumonia. Furthermore, the impact of the post COVID-19 hospitalisation events described in this study upon the NHS in England is substantial. Future work should investigate any association between non-hospitalised SARS-CoV-2 infection and these outcomes, and quantify any likely population level impact.

## Data and software availability

### Underlying data

All data were linked, stored and analysed securely within the OpenSAFELY platform ( https://opensafely.org/). Data include pseudonymized data such as coded diagnoses, medications and physiological parameters. No free text data are included. All code is shared openly for review and re-use under MIT open license. Detailed pseudonymized patient data are potentially re-identifiable and therefore not shared.

For security and privacy reasons, OpenSAFELY is very different to other approaches for EHR data analysis. The platform does not give researchers unconstrained access to view large volumes of pseudonymised and disclosive patient data, either via download or via a remote desktop. Instead we have produced a series of open source tools that enable researchers to use flexible, pragmatic, but standardised approaches to process raw electronic health records data into “research ready” datasets, and to check that this has been done correctly, without needing to access the patient data directly. Using this data management framework we also generate bespoke dummy datasets. These dummy datasets are used by researchers to develop analysis code in the open, using GitHub. When their data management and data analysis scripts are capable of running to completion, and passing all tests in the OpenSAFELY framework, they are finally sent through to be executed against the real data inside the secure environment, using the OpenSAFELY jobs runner, inside a container using Docker, without the researcher needing access to that raw potential disclosive pseudonymised data themselves. The non-disclosive summary results output tables, logs, and graphs are then manually reviewed, as in other systems, before release.

As part of building that resource for the community, we are working with NHS England to cautiously on-board a small number of external pilot users to develop their analyses on OpenSAFELY. This process is described in further detail on our webpage.

### Extended data

Analysis code available from: https://github.com/opensafely/post-covid-outcomes-research

Archived analysis code at time of publication: https://doi.org/10.5281/zenodo.6475808 ^28^

Data are available under the terms of the Creative Commons Attribution 4.0 International license (CC-BY 4.0).

Zenodo: Extended data: Supplemental tables and figures for post-covid paper. https://doi.org/10.5281/zenodo.6475823 ^29^

This project contains the following extended data:

- Supplemental Material.pdf

Data are available under the terms of the Creative Commons Attribution 4.0 International license (CC-BY 4.0).
