# Supplementary material for: Rates of serious clinical outcomes in survivors of hospitalisation with COVID-19 in England: a descriptive cohort study within the OpenSAFELY platform
Source: Wellcome Open Res. 2022 Apr 29;7:142. [Version 1] doi: 10.12688/wellcomeopenres.17735.1 (PMC10285340; doi:10.12688/wellcomeopenres.17735.1)
Supplement: Peer review history

# Peer review: Reviewer response for version 1

**DOI:** 10.21956/wellcomeopenres.19626.r56249

**Published:** 2023-06-21

**Authors:** Daniel R. Morales

**Affiliations:** 1 Division of Population Health and Genomics, School of Medicine, Ninewells Hospital and Medical School, University of Dundee, Dundee, UK

The authors have performed an interesting and robust study.

 Minor comments for consideration would include:

1. Clarifying whether outcomes were identified from hospitalization using codes in any position or as a primary reason for admission. 
2. In the discussion section it states "especially in light of the observed higher in-hospital mortality rate for hospitalised COVID-19 patients compared to patients hospitalised with pneumonia." The authors could consider adding a specific example from the results as to why this might be important (e.g. the lower SHR for heart failure if people with covid19 & pre-existing heart failure were more likely to die). 

Is the work clearly and accurately presented and does it cite the current literature?

Yes

If applicable, is the statistical analysis and its interpretation appropriate?

Yes

Are all the source data underlying the results available to ensure full reproducibility?

Yes

Is the study design appropriate and is the work technically sound?

Yes

Are the conclusions drawn adequately supported by the results?

Yes

Are sufficient details of methods and analysis provided to allow replication by others?

Yes

Reviewer Expertise:

Primary care, clinical epidemiology

I confirm that I have read this submission and believe that I have an appropriate level of expertise to confirm that it is of an acceptable scientific standard.

# Peer review: Reviewer response for version 1

**DOI:** 10.21956/wellcomeopenres.19626.r50352

**Published:** 2022-05-31

**Authors:** Christian Fynbo Christiansen, Simon Kok Jensen

**Affiliations:** 1 Department of Clinical Epidemiology, Aarhus University Hospital, Aarhus, Denmark

This well-written manuscript by Tazare *et al.* describes the rates of seven clinical outcomes in patients surviving a hospitalization with COVID-19 and compares these to rates in a general population cohort and a cohort surviving a hospitalization with pneumonia. The study found that patients with COVID-19 have higher rates of adverse outcomes when compared with the general population, but similar rates of adverse outcomes when compared to patients surviving a hospitalization with pneumonia.

 The study is well-designed, and the findings highlight the importance of the selection of comparison cohort when studying the prognosis of COVID-19.

 Specific comments to be addressed:

  

- It could be described why these seven outcomes were selected. Most were acute events, but it also includes chronic disease (Type 2 Diabetes mellitus (T2DM) and heart failure). Did the authors consider including respiratory conditions?
- The increased rate of T2DM in patients with COVID-19 compared with patients with pneumonia is mentioned in the abstract, but the rate of 15.2 per 1,000 person-years cannot be found in Table 3. The rate for PE is also higher in COVID-19 patients while there is a lower rate for heart failure. This could also be mentioned in the abstract.

- The comparison with both a general population cohort and a pneumonia cohort is an important strength of the study. The known association between influenza and cardiovascular events is mentioned in the introduction and it could be considered also to include a third comparison cohort of patients hospitalized with influenza. This would allow for a comparison of adverse outcomes between a common and well-known viral infection and COVID-19.
- In “outcomes and follow-up”: “AKI” should be written out the first time it is used.
- The rationale for using a Fine and Grey regression could be mentioned and the challenges with the interpretation of subdistributional hazards could be discussed (Austin and Fine, 2017 ^1^).
- In the primary analysis patients with a recorded outcome within three months before the index are excluded. Is it possible that this could result in including patients with prevalent disease, e.g., heart failure or T2DM, if that patient has not seen a physician during the three months before index? This may contribute to the high rate of heart failure after discharge (Table 3). Have the authors considered doing a sensitivity analysis only including incident outcomes with e.g. a 5 year look-back period?

Minor comment:

- AKI should be spelled out in the abstract and in the “Outcomes and follow-up” in the methods section.

Is the work clearly and accurately presented and does it cite the current literature?

Yes

If applicable, is the statistical analysis and its interpretation appropriate?

Yes

Are all the source data underlying the results available to ensure full reproducibility?

Yes

Is the study design appropriate and is the work technically sound?

Yes

Are the conclusions drawn adequately supported by the results?

Yes

Are sufficient details of methods and analysis provided to allow replication by others?

Yes

Reviewer Expertise:

Clinical epidemiology.

We confirm that we have read this submission and believe that we have an appropriate level of expertise to confirm that it is of an acceptable scientific standard, however we have significant reservations, as outlined above.

## References

[1] : Practical recommendations for reporting Fine-Gray model analyses for competing risk data. *Stat Med* .2017;36(27) : 10.1002/sim.7501 4391-4400 10.1002/sim.7501 28913837PMC5698744

# Peer review: Reviewer response for version 1

**DOI:** 10.21956/wellcomeopenres.19626.r50605

**Published:** 2022-05-30

**Authors:** Ruth Brauer

**Affiliations:** 1 Research Department of Practice and Policy, School of Pharmacy, University College London, London, UK

This is an informative study, clearly describing the elevated risk of 7 pre-selected outcomes after discharge from a hospital stay during which patients were diagnosed with Covid-19.

 My only concern is that 5 out of the 7 outcomes (a somewhat random selection) could be recurrent events. According to Table 1, a high proportion of patients with Covid-19 had a history of heart failure, MI and stroke, particularly compared to the general population. The risk of a recurrent event is higher than the risk of an incident event, so this may have affected the age, gender and region adjusted results somewhat. The post-hoc analyses address this worry to some extent, but the implications of measuring recurrent vs incident events should still be mentioned in the Discussion.

 Minor comments:

- Abbreviations, such as AKI (abstract) and SHR, appear without explanation. Likewise, none of the tables explain any of the abbreviations used. 
- The introduction does not make it clear why the 7 specific outcomes were chosen. Whilst some are mentioned, others are not (i.e. T2DM).
- Table 4 shows that diabetes was a covariate in the fully adjusted analyses, but I assume that for the T2DM analyses this covariate was omitted? Maybe this information can be added.

Is the work clearly and accurately presented and does it cite the current literature?

Yes

If applicable, is the statistical analysis and its interpretation appropriate?

Yes

Are all the source data underlying the results available to ensure full reproducibility?

Yes

Is the study design appropriate and is the work technically sound?

Partly

Are the conclusions drawn adequately supported by the results?

Yes

Are sufficient details of methods and analysis provided to allow replication by others?

Yes

Reviewer Expertise:

Pharmaco-epidemiology; Electronic health records research

I confirm that I have read this submission and believe that I have an appropriate level of expertise to confirm that it is of an acceptable scientific standard.
